# Physiological Stress Signatures of Waterborne Glyphosate Exposure in *Apostichopus japonicus*: Insights for Aquatic Ecotoxicology

**DOI:** 10.3390/toxics14040282

**Published:** 2026-03-26

**Authors:** Jingchun Sun, Shaoping Kuang, Hongsheng Yang

**Affiliations:** 1 College of Environment and Safety Engineering, Qingdao University of Science and Technology, Qingdao 266042, China; sunjingchun@qdio.ac.cn; 2 Laboratory of Marine Ecology and Environmental Sciences, Institute of Oceanology, Chinese Academy of Sciences, Qingdao 266000, China; 3 Laboratory for Marine Ecology and Environmental Science, Qingdao Marine Science and Technology Center, Qingdao 266237, China; 4 State Key Laboratory of Breeding Biotechnology and Sustainable Aquaculture, Institute of Oceanology, Chinese Academy of Sciences, Qingdao 266000, China

**Keywords:** glyphosate, *Apostichopus japonicus*, waterborne exposure, tissue distribution, oxidative stress, digestive enzymes, gut microbiota

## Abstract

Glyphosate is a widely used herbicide with increasing concern regarding its non-target impacts in coastal ecosystems and mariculture species. Here, we profiled acute physiological stress signatures of waterborne glyphosate exposure in the sea cucumber *Apostichopus japonicus*, integrating measured exposure concentrations, tissue residues, digestive and oxidative/innate immune biomarkers, and gut microbiota. After 24 h exposure, measured waterborne glyphosate confirmed the intended gradient (0.09 ± 0.02, 1.26 ± 0.09, and 4.49 ± 1.12 mg/L for low-, medium-, and high-dose treatments, respectively), and overt stress phenotypes with mortality occurred only at the high dose (36.67%), enabling separation of high-dose survivors (HS) and high-dose dead (HD) for downstream analyses. Tissue measurements showed low/background levels in controls, with compartment-specific distribution: the respiratory tree exhibited higher burdens at the medium dose, whereas coelomic fluid showed the highest burdens in HS at the 24 h endpoint. Functionally, most intestinal digestive enzymes were unchanged, but trypsin activity was consistently suppressed across exposed groups (*p* < 0.05). In coelomic fluid, oxidative stress responses were evident, with elevated MDA (L and M), reduced CAT (L, M, and HS), and reduced GSH-PX in HS (all *p* < 0.05), while SOD, GR, and lysozyme showed no significant changes. Gene sequencing of 16S rRNA (*n* = 3 per group) revealed significant shifts in community diversity/evenness (Shannon *p* = 0.0497; Simpson *p* = 0.0484) and beta diversity (PCo1 = 30.08%, PCo2 = 26.30%; PERMANOVA F = 1.816, *p* = 0.008), with LEfSe indicating discriminative taxa associated with exposure/outcomes. Collectively, these multi-level endpoints define an acute glyphosate stress signature in *A. japonicus*, linking internal dose distribution to oxidative disruption, impaired intestinal proteolysis, and microbiome restructuring.

## 1. Introduction

Glyphosate (N-(phosphonomethyl)glycine) is the most widely used herbicide globally and remains central to modern weed control because of its broad-spectrum efficacy and systemic action in plants [1]. Although its primary mode of action targets the shikimate pathway in plants, extensive application and multiple release routes—including spray drift, surface runoff, leaching, wastewater discharge, and industrial effluents—have made glyphosate and its main metabolite aminomethylphosphonic acid (AMPA) ubiquitous contaminants of aquatic environments [2,3,4,5,6]. In recent years, the environmental debate around glyphosate has expanded beyond terrestrial risk to include aquatic exposure scenarios, mixture effects from commercial formulations, and uncertainty in hazard characterization across taxa and life stages [2,3,4,7]. Regulatory evaluations have also highlighted the need for refined exposure and effect datasets that better represent realistic environmental conditions and non-target species diversity [8,9,10].

The presence of glyphosate and AMPA in surface waters, groundwater, and drinking-water sources has been repeatedly documented across regions with intensive agriculture and dense human activities [5,6,11,12]. Recent monitoring continues to report measurable concentrations in freshwater bodies and watersheds influenced by cropland runoff and land-use patterns [11,12]. However, translating occurrence data into ecological risk remains challenging because (i) environmental concentrations can be episodic and highly variable; (ii) toxicity may depend on formulation additives (e.g., surfactants), water chemistry, and temperature; and (iii) sensitive endpoints may appear before overt mortality [2,3,7,8]. Analytical complexity further contributes to uncertainty: glyphosate’s high polarity, zwitterionic nature, and strong metal/particle interactions complicate extraction and quantification in natural waters, motivating continued optimization of chromatographic workflows (often involving derivatization and fluorescence or MS detection) for robust monitoring and exposure confirmation [13,14,15].

A growing body of aquatic ecotoxicology research indicates that glyphosate exposure can induce sublethal disturbances in fish and invertebrates, including neurotoxicity, oxidative stress, immunotoxicity, and metabolic dysregulation, even when acute lethality is not observed [2,3,4,7,16,17,18]. Reviews synthesizing evidence in aquatic vertebrates emphasize that biochemical and physiological biomarkers—such as antioxidant enzymes (e.g., SOD, CAT, and GPx), lipid peroxidation products, acetylcholinesterase activity, and immune-related enzymes—are frequently responsive to herbicide exposure and can provide mechanistic signals of stress and adaptive costs [4,7,17,18]. For example, a recent study in freshwater crayfish demonstrated that glyphosate exposure altered oxidative status, neurochemical markers, and gut microbial composition, supporting the idea that multi-system responses may co-occur under waterborne exposure [16]. Such findings reinforce that risk assessment based solely on survival may underestimate ecologically relevant impacts on growth, digestion, immunity, and disease susceptibility [2,3,7,17,18].

In parallel, the intestinal microbiome has emerged as a sensitive and functionally meaningful interface linking environmental contaminants to host physiology in aquatic organisms [19]. The gut microbial community supports nutrient assimilation, energy harvest, immune homeostasis, xenobiotic biotransformation, and mucosal barrier maintenance; therefore, pollutant-driven dysbiosis can translate into downstream consequences for digestive capacity and systemic resilience [19]. From an ecotoxicology perspective, microbiome metrics can complement classical enzyme biomarkers by providing community-level signatures that integrate exposure intensity, host stress, and ecological interactions with pathogens [19]. This is particularly relevant for aquaculture species, where environmental contamination and husbandry stressors can synergize to shape gut ecology and health outcomes [20].

Sea cucumbers are high-value benthic invertebrates that contribute to sediment bioturbation and nutrient cycling, and many species underpin intensive coastal aquaculture in Asia [21]. The sea cucumber *Apostichopus japonicus* is one of the most economically important holothurians; its farming systems are widely distributed in nearshore coastal zones in China, where they may be exposed to land-based inputs and episodic contamination events [22]. Despite the expanding literature on holothurian physiology, immunity, and environmental stress responses [22,23,24], information remains limited on how herbicides—especially glyphosate, which has been extensively evaluated in regulatory risk assessments [25,26]—affect sea cucumber health under waterborne exposure scenarios. Existing work in *A. japonicus* and related contexts shows that chemical and environmental stressors can modulate non-specific immune parameters and oxidative status, underscoring the utility of enzyme-based biomarkers for diagnosing stress in this species [22,23,27,28]. Stress responses in sea cucumbers can arise from multiple sources, including environmental fluctuations (e.g., temperature, salinity, and dissolved oxygen), husbandry-related disturbances, and exposure to chemical toxicants. Distinguishing among these stressors is important because environmental stress may interact with toxicant exposure and potentially amplify physiological responses. Moreover, recent microbiome-focused studies in *A. japonicus* demonstrate that stress can reshape intestinal communities and functional potential, providing an additional layer of evidence for sublethal impacts that may not be captured by traditional endpoints alone [22,23,29]. Because gut microbial structure is also associated with quality traits and metabolism in sea cucumbers, perturbations may have implications extending from animal health to product quality [30].

Against this backdrop, profiling physiological stress “signatures” under waterborne glyphosate exposure in *A. japonicus* is timely for aquatic ecotoxicology and coastal aquaculture management. Integrating exposure confirmation (internal residue signals) with digestive enzyme activities, immune/antioxidant enzyme responses, and 16S rRNA-based gut microbiota analysis can provide a more comprehensive assessment of how a polar herbicide perturbs host function across multiple biological layers. Such an approach helps bridge monitoring data and biological effects by linking waterborne exposure to measurable physiological alterations in a representative mariculture invertebrate, thereby supporting evidence-based risk evaluation and water-quality protection strategies in nearshore farming regions [2,3,8].

## 2. Materials and Methods

### 2.1. Animals and Acclimation

Sea cucumbers (*Apostichopus japonicus*) were obtained from a commercial farm in Rushan, Shandong, China. The animals had body weights ranging from 50 to 100 g at the start of the experiment. Because they were purchased from a commercial farm, the precise age of the individuals was not available from the supplier. The animals were acclimated in 65 L tanks for 3 days before exposure. The exposure water consisted of filtered seawater prepared under laboratory conditions, which was continuously aerated and maintained at the experimental temperature during acclimation and exposure.

### 2.2. Experimental Design and Waterborne Exposure

A total of 120 healthy sea cucumbers (*Apostichopus japonicus*) were randomly assigned to four exposure groups: control (C) and three waterborne glyphosate treatments (L, low dose; M, medium dose; H, high dose). Each group contained three independent replicate buckets (*n* = 3 buckets per group), and each bucket housed 10 individuals (10 sea cucumbers per bucket; 30 individuals per group). Glyphosate exposure solutions were prepared by dissolving glyphosate reagent powder (catalog no. N817057-100g; Macklin Biochemical Co., Ltd., Shanghai, China; purity 95%) to prepare a stock solution, which was subsequently diluted to the target concentrations for the low-, medium-, and high-dose treatments. Exposure lasted for 24 h. At the 24 h endpoint, the high-dose group was further stratified by outcome into high-dose survivors (HS) and high-dose dead (HD) for downstream endpoint analysis and figure presentation.

### 2.3. Sampling Strategy and Sample Sizes for Different Endpoints

At the 24 h endpoint, sea cucumbers were dissected on ice. For glyphosate quantification and physiological/biochemical assays, five individuals were randomly sampled from each of the C, L, and M groups (*n* = 5 per group). For the high-dose treatment, samples were collected separately from high-dose survivors (HS) and dead individuals (HD), with five individuals analyzed per subgroup whenever applicable (*n* = 5 for HS; *n* = 5 for HD). For 16S rRNA gene sequencing, intestinal content samples were collected from three individuals per group/subgroup (C, L, M, HS, and HD; *n* = 3 per group).

Samples of body wall, respiratory tree, intestine, and coelomic fluid were collected for glyphosate residue analysis. Intestinal tissue was used for digestive enzyme assays, coelomic fluid was used for immune and antioxidant biomarker analyses, and intestinal contents were collected under sterile conditions for gut microbiota analysis. All tissues and fluids were immediately processed or stored at −80 °C until further analysis.

### 2.4. Glyphosate Analysis

Glyphosate concentrations were determined using a derivatization-based method according to SN/T 1923-2007 [31]. Biological samples for glyphosate determination were processed on a wet-weight basis. No drying step was applied prior to analysis; instead, the samples were directly subjected to the extraction procedure. Briefly, 2.5 g of each biological sample was weighed, and 25 mL of water was added. The mixture was sonicated for 30 min, followed by centrifugation. An aliquot of the supernatant was extracted with chloroform, and glyphosate was derivatized with a borax and fluorenylmethoxy reagent. After derivatization, the sample was extracted with ethyl acetate and analyzed by HPLC with fluorescence detection. Water samples were collected at the 24 h endpoint to analytically verify the actual exposure concentrations.

### 2.5. Digestive Enzyme Assays

Digestive enzymes in intestinal tissue, including alkaline phosphatase (ALP), acid phosphatase (ACP), cellulase (CL), trypsin, lipase (LPS), and amylase (AMS), were quantified using commercial assay kits (Nanjing Jiancheng Bioengineering Institute, Nanjing, China) according to the manufacturers’ instructions. Briefly, ~0.1 g of fresh intestine tissue was homogenized in 1× PBS buffer (I010-1-1; Nanjing Jiancheng) using a bead mill (65 Hz, 10 min) under cold conditions. After centrifugation (3500 rpm, 15 min, 4 °C), the supernatant was collected for enzyme activity assays. Enzyme activities were normalized to protein content where required (e.g., U/mg protein) following the kit manuals and the corresponding protein determination procedure.

### 2.6. Immune and Antioxidant Biomarkers

Innate immune and oxidative stress-related biomarkers in coelomic fluid, including superoxide dismutase (SOD), catalase (CAT), glutathione peroxidase (GSH-PX), glutathione reductase (GR), malondialdehyde (MDA), and lysozyme (LZM), were measured using commercial assay kits (Nanjing Jiancheng Bioengineering Institute, Nanjing, China) following the manufacturers’ protocols. Fresh coelomic fluid aliquots were mixed with pre-cooled extraction buffer (1:10, *v*/*v*), homogenized (2500 rpm, 10 min), and centrifuged (3500 rpm, 10 min, 4 °C). Supernatants were used for subsequent assays. Units and normalization followed kit manuals (e.g., U/mL, U/L, nmol/mL, etc., as shown in each panel).

### 2.7. Gut Microbiota Analysis

The gut microbiota was assessed using 16S rRNA gene sequencing. DNA was ex-tracted from the gut content of each individual using the HiPure Stool DNA Kit (Magen Biotechnology Co., Ltd., Guangzhou, China) following the manufacturer’s instruc-tions. Intestinal content samples were collected under sterile conditions and immedi-ately stored at −80 °C until DNA extraction. No drying step was applied prior to mi-crobiota analysis. The V3–V4 region of the 16S rRNA gene was amplified using primers 341F (CCTACGGGNGGCWGCAG) and 806R (GGACTACHVGGGTATCTAAT). PCR amplification was performed using Q5 High-Fidelity DNA Polymerase (New England Biolabs, Ipswich, MA, USA), and the PCR products were purified with AMPure XP beads (Beckman Coulter, Brea, CA, USA). DNA concentration was quantified using a Qubit 3.0 Fluorometer (Thermo Fisher Scientific, Waltham, MA, USA), and real-time PCR was performed on an ABI StepOnePlus Real-Time PCR System (Thermo Fisher Scientific, Waltham, MA, USA). Libraries were sequenced on an Illumina NovaSeq 6000 platform (Illumina, San Diego, CA, USA) using PE250 mode. Sequence data were processed and analyzed using the QIIME2 pipeline for taxonomic classification and diversity analysis.

### 2.8. Statistical Analysis

All data are presented as mean ± standard deviation (SD). Glyphosate concentrations in different tissues were analyzed using an ordinary two-way ANOVA, with treatment group (control group, low-concentration treatment group, medium-concentration treatment group, high-concentration survivor group, and high-concentration dead group) as the row factor and tissue type (body wall, intestine, respiratory tree, and coelomic fluid) as the column factor. The analysis first evaluated the main effects of treatment group and tissue type, as well as the interaction between these two factors. When significant main effects or interactions were detected, Bonferroni’s multiple comparisons test was performed for pairwise comparisons among relevant cell means. A *p* value < 0.05 was considered statistically significant. All statistical analyses were conducted using GraphPad Prism version 8.0 (GraphPad Software, San Diego, CA, USA).

## 3. Results

### 3.1. Acute Phenotypic Responses and Exposure Verification in Water

**Figure 1:**
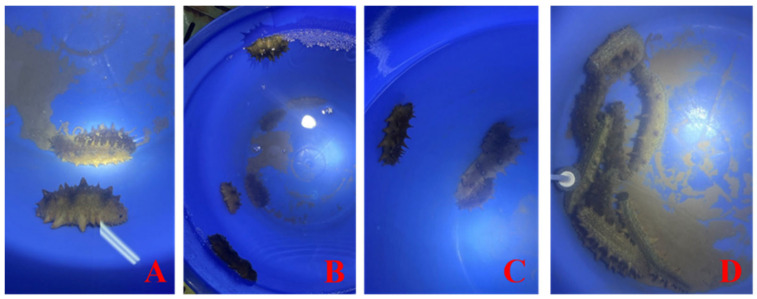
Representative phenotypes of *Apostichopus japonicus* after 24 h exposure. (**A**) Control group; (**B**) low-concentration group; (**C**) medium-concentration group; (**D**) high-concentration group. Measured waterborne glyphosate concentrations confirmed the intended exposure gradient. The control seawater contained only background-level glyphosate (practically not detected), whereas glyphosate concentrations increased across treatments, reaching 0.09 ± 0.02 mg/L (L), 1.26 ± 0.09 mg/L (M), and 4.49 ± 1.12 mg/L (H).

**Figure 2:**
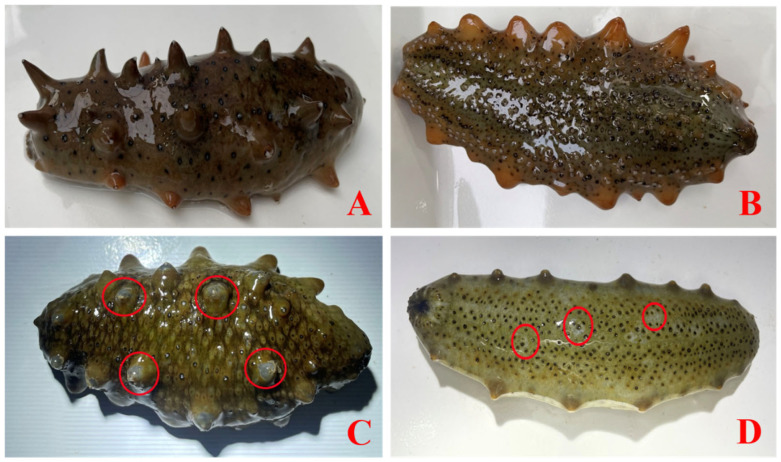
Body-wall and ventral surface conditions of *Apostichopus japonicus* after 24 h exposure. (**A**) Dorsal view of the control group; (**B**) ventral view of the control group; (**C**) dorsal view of the high-concentration dead group; (**D**) ventral view of the high-concentration dead group. The circle indicates the region where early tissue decay was observed in the sea cucumber.

After 24 h of waterborne glyphosate exposure, no mortality or apparent skin lesions (“skin peeling”) were observed in the control (C), low-dose (L), or medium-dose (M) groups. In contrast, individuals in the high-dose treatment displayed marked acute stress phenotypes, including skin peeling, tentacle contraction, and body-wall rigidity, accompanied by mortality (36.67%) (Figure 1 and Figure 2). Therefore, the high-dose treatment was further separated into a high-dose dead (HD) group and a high-dose survivor (HS) group for downstream analyses.

### 3.2. Tissue Distribution of Glyphosate After Waterborne Exposure

**Figure 3:**
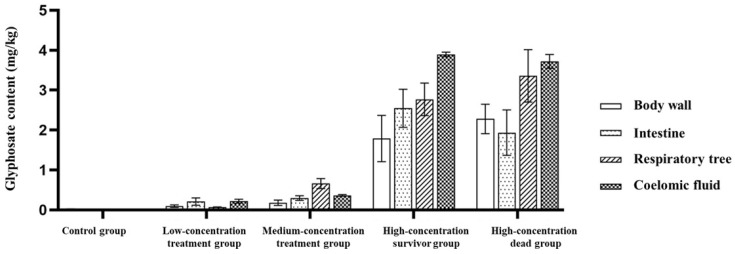
Glyphosate distribution in different tissues of *Apostichopus japonicus* under different exposure conditions. Data are presented as mean ± SD. Statistical significance was evaluated using ordinary two-way ANOVA with treatment group and tissue type as the two factors, followed by Bonferroni’s multiple comparisons test.

Glyphosate concentrations differed significantly among treatment groups and tissues (Figure 3). Ordinary two-way ANOVA revealed a highly significant interaction between treatment group and tissue type (F(12, 40) = 8.111, *p* < 0.0001), indicating that tissue-specific glyphosate distribution patterns depended on exposure condition. In addition, both the treatment group factor (F(4, 40) = 293.0, *p* < 0.0001) and the tissue factor (F(3, 40) = 21.93, *p* < 0.0001) were significant.

In the control group, glyphosate concentrations remained low across all tissues. In the low-concentration treatment group, glyphosate levels increased slightly, but tissue-specific differences remained limited. In the medium-concentration treatment group, the respiratory tree showed a higher accumulation tendency than the other tissues. In the high-concentration survivor group, glyphosate concentrations in the respiratory tree and coelomic fluid were markedly elevated compared with those in the body wall, and coelomic fluid was also higher than in the intestine. In the high-concentration dead group, glyphosate burdens remained high across tissues, with coelomic fluid showing the highest accumulation, while the body wall remained comparatively lower.

Bonferroni’s multiple comparisons further supported that tissue differences became more pronounced under medium- and high-exposure conditions, whereas tissue variation was relatively small in the control and low-concentration groups (Figure 3).

### 3.3. Digestive Enzyme Responses in the Intestine

**Figure 4:**
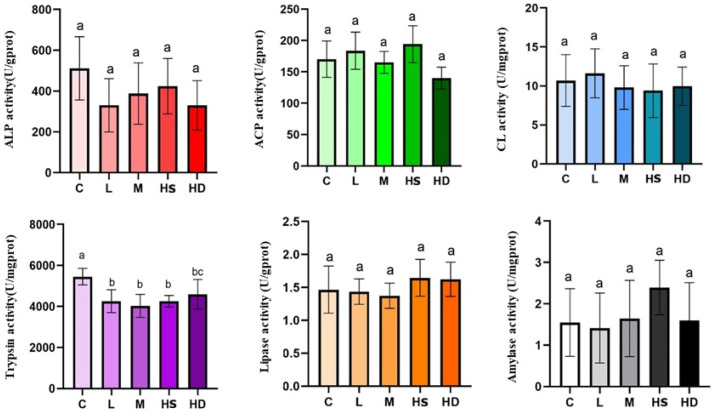
Digestive enzyme activities in intestinal tissue. Activities of ALP, ACP, CL, trypsin, LPS, and AMS in intestinal tissue after 24 h exposure. Data are presented as mean ± SD. Different letters indicate significant differences among groups (one-way ANOVA followed by Tukey’s HSD post hoc test, or Kruskal–Wallis test with appropriate multiple-comparison correction, as appropriate, *p* < 0.05). Units follow kit manuals and are shown on each panel (e.g., U/mg protein). Sample size: *n* = 5 for C, L, and M; *n* = 5 for HS and HD whenever applicable. Groups: C, control; L, low dose; M, medium dose; HS, high-dose survivors; HD, high-dose dead.

Most assayed digestive enzymes—including alkaline phosphatase (ALP), acid phosphatase (ACP), cellulase (CL), lipase (LPS), and amylase (AMS)—showed no significant differences compared with the control under acute glyphosate exposure. In contrast, trypsin activity was significantly reduced in the L, M, HS, and HD groups relative to the control (*p* < 0.05), indicating that acute waterborne glyphosate exposure can rapidly perturb intestinal proteolytic capacity even when several other digestive functions appear unchanged (Figure 4).

### 3.4. Innate Immune and Oxidative Stress Enzymes in Coelomic Fluid

**Figure 5:**
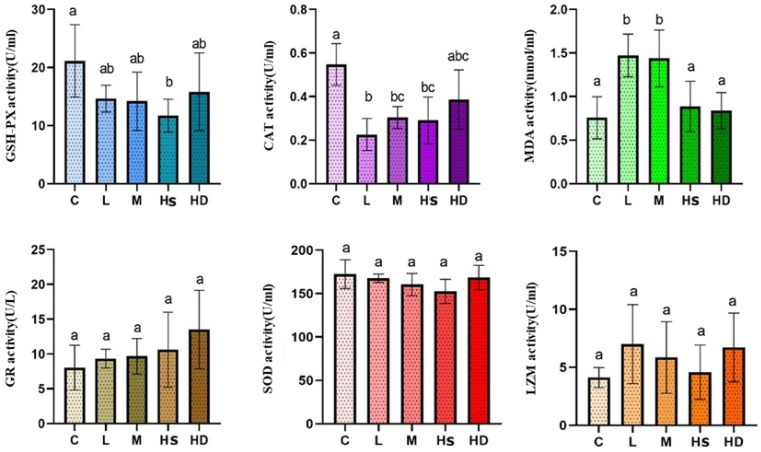
Immune and oxidative stress-related biomarkers in coelomic fluid. Levels/activities of SOD, CAT, GSH-PX, GR, MDA, and LZM in coelomic fluid after 24 h exposure. Data are presented as mean ± SD. Different letters indicate significant differences among groups (one-way ANOVA followed by Tukey’s HSD post hoc test, or Kruskal–Wallis test with appropriate multiple-comparison correction, as appropriate, *p* < 0.05). Units follow kit manuals and are shown on each panel (e.g., U/mL, U/L, nmol/mL). Sample size: *n* = 5 for C, L, and M; *n* = 5 for HS and HD whenever applicable. Groups: C, control; L, low dose; M, medium dose; HS, high-dose survivors; HD, high-dose dead.

Across immune/oxidative endpoints, several enzymes (e.g., superoxide dismutase, SOD; lysozyme, LZM; glutathione reductase, GR) showed no significant differences from the control under acute exposure. However, lipid peroxidation and antioxidant defense markers responded sensitively. Specifically, MDA was elevated in the L and M groups relative to the control (*p* < 0.05), while CAT activity was reduced in the L, M, and HS groups (*p* < 0.05). GSH-PX also decreased in the HS group compared with the control (*p* < 0.05). Collectively, these results indicate that acute waterborne glyphosate stress induces a measurable oxidative stress signature in coelomic fluid, even when parts of the innate immune enzyme panel remain statistically unchanged (Figure 5).

### 3.5. Gut Microbiota Shifts Revealed by 16S rRNA Gene Sequencing

#### 3.5.1. Community Composition Overview

**Figure 6:**
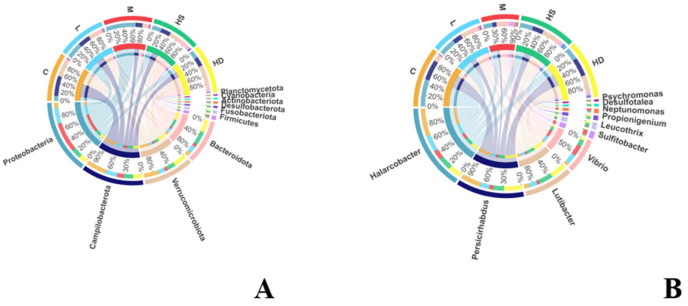
Relative abundance profiles of the top-10 taxa at (**A**) phylum and (**B**) genus levels across groups (*n* = 3 per group). Taxa are ranked by overall relative abundance across all samples; only the ten most abundant taxa are shown in each panel. Group abbreviations are consistent with the main text (C, L, M, HS, and HD).

Top-10 relative abundance profiles indicated that the gut microbiota was dominated by *Proteobacteria*, *Campylobacterota*, *Verrucomicrobiota*, and *Bacteroidota* at the phylum level, while *Halarcobacter*, *Persicirhabdus*, *Lutibacter*, and *Vibrio* were among the dominant genera across groups (*n* = 3 per group) (Figure 6A,B).

#### 3.5.2. Alpha Diversity Patterns

**Figure 7:**
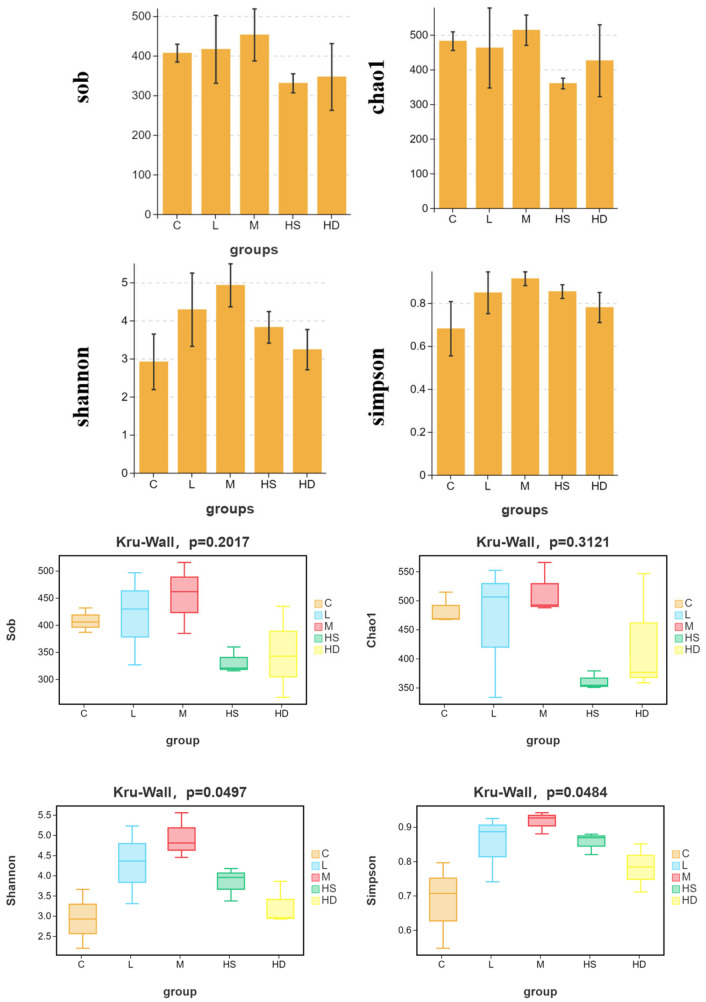
Alpha diversity indices of gut microbiota across groups. Alpha diversity shown as Sob (observed OTUs), Chao1, Shannon, and Simpson across groups (*n* = 3 per group). Boxplots display median and IQR; whiskers indicate dispersion. Overall group differences were evaluated using the Kruskal–Wallis test (Sob: *p* = 0.2017; Chao1: *p* = 0.3121; Shannon: *p* = 0.0497; Simpson: *p* = 0.0484). Groups: C, control; L, low dose; M, medium dose; HS, high-dose survivors; HD, high-dose dead.

Alpha diversity analysis (*n* = 3 per group) showed that richness-related metrics (Sob, i.e., observed OTUs, and Chao1) did not differ significantly among groups (Kruskal–Wallis: Sob, *p* = 0.2017; Chao1, *p* = 0.3121). In contrast, diversity/evenness-related indices displayed significant overall group effects, with Shannon (*p* = 0.0497) and Simpson (*p* = 0.0484) differing among treatments (Figure 7), suggesting that acute exposure was associated primarily with changes in community diversity/evenness rather than consistent shifts in richness alone.

#### 3.5.3. Beta Diversity and PERMANOVA

**Figure 8:**
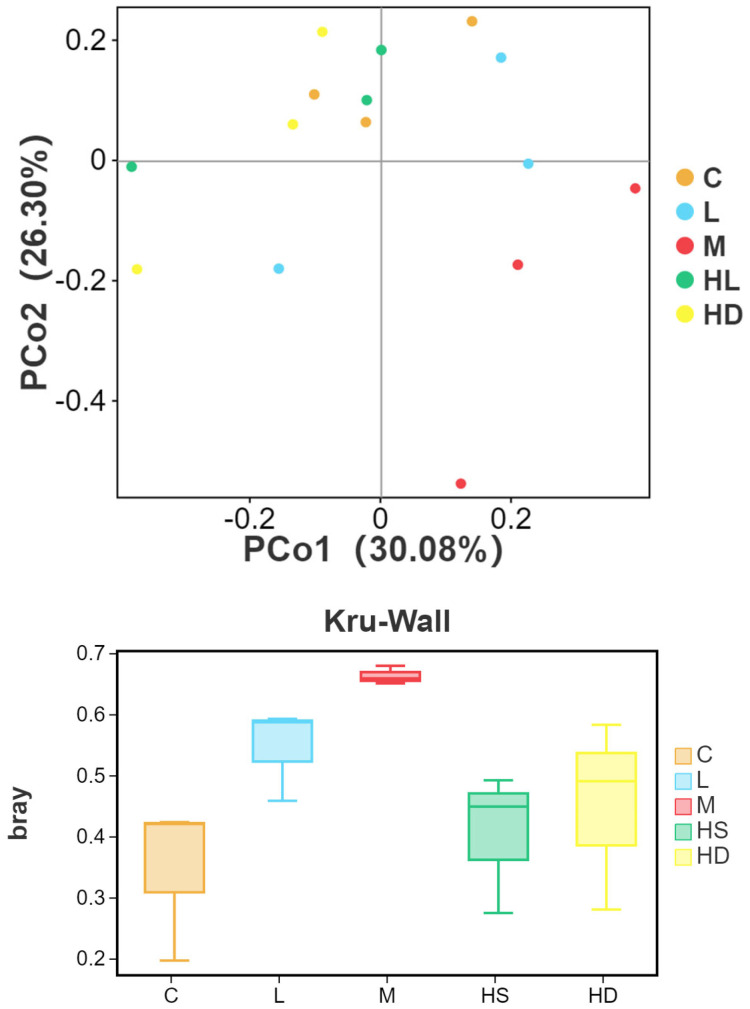
PCoA of Bray–Curtis dissimilarities showing beta diversity patterns. PCoA based on Bray–Curtis distances of OTU profiles across all samples. The first two axes explained 30.08% (PCo1) and 26.30% (PCo2) of total variation. Community composition differed significantly among groups by PERMANOVA (F = 1.816, *p* = 0.008; 999 permutations). Groups: C, control; L, low dose; M, medium dose; HS, high-dose survivors; HD, high-dose dead.

Bray–Curtis-based beta diversity analysis supported treatment-associated community shifts. PCoA showed that PCo1 and PCo2 explained 30.08% and 26.30% of the total variation, respectively. A one-way PERMANOVA confirmed significant differences in community composition among groups (F = 1.816, *p* = 0.008; 999 permutations) (Figure 8).

#### 3.5.4. Differential Taxa Identified by LefSe

**Figure 9:**
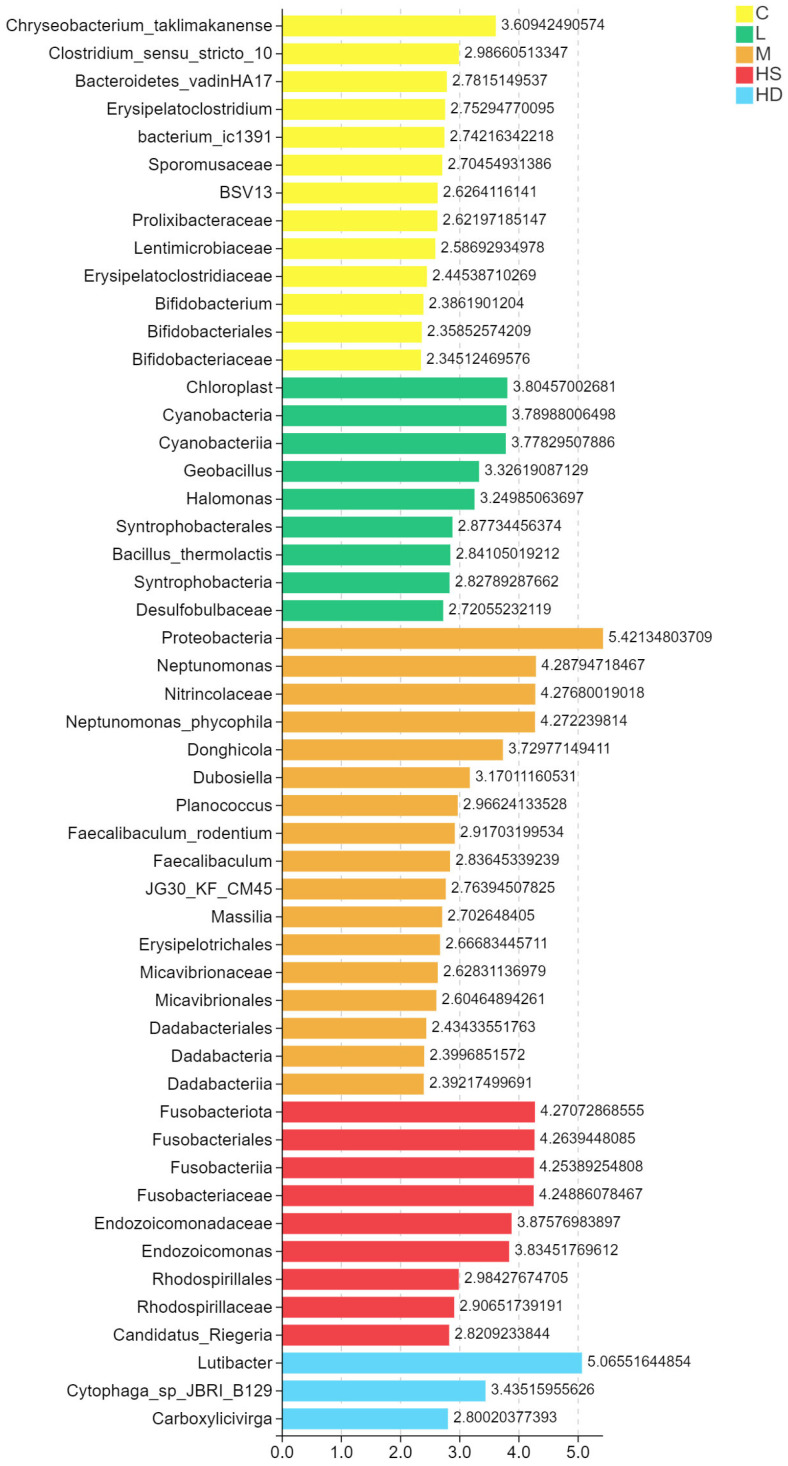
LEfSe-identified discriminative taxa among groups. LDA score bar plot showing taxa differentially enriched in each group (LDA threshold as defined in Section 2). Groups: C, control; L, low dose; M, medium dose; HS, high-dose survivors; HD, high-dose dead.

LEfSe analysis identified discriminative taxa associated with exposure treatments/outcomes. Differentially enriched lineages were visualized by an LDA score plot (Figure 9), providing candidate microbial signatures distinguishing control and exposed groups.

## 4. Discussion

### 4.1. Exposure Characterization and Environmental Relevance

In this study, waterborne glyphosate was not detected in the control water, whereas measured concentrations increased stepwise across the low, medium, and high treatments (0.09, 1.26, and 4.49 mg/L). This confirms that the exposure gradient was achieved analytically rather than being inferred from nominal dosing, which is essential for interpreting concentration–response patterns in aquatic toxicology [13,15]. Although these mg/L levels exceed most background concentrations reported for rivers and coastal waters, episodic pulses can occur near agricultural runoff inputs, drainage channels, and point-source releases, and short-term high exposures are often used to define hazard thresholds and stress signatures in non-target organisms [3,4,5,6]. Importantly, glyphosate risk in aquatic systems is frequently discussed in the context of formulations (glyphosate-based herbicides, GBHs) and co-occurring stressors (temperature, salinity, nutrient load), which can amplify sublethal effects even at “environmentally realistic” concentrations [5,32]. Therefore, the present acute design can be viewed as a mechanistic stress test that clarifies early-warning endpoints (redox imbalance, digestive inhibition, and microbiome shifts) relevant to both ecotoxicology and aquaculture health management [16,33,34].

### 4.2. Acute Lethality Indicates Limited Tolerance at the Highest Exposure

The mortality observed in the high treatment indicates that *A. japonicus* exhibits limited tolerance to abrupt increases in waterborne glyphosate under the tested conditions. Similar acute-to-subacute outcomes (survival reduction co-occurring with oxidative stress and metabolic disturbance) have been documented across aquatic taxa exposed to glyphosate/GBHs, with variability driven by species traits, developmental stage, and environmental context [4,5,32]. Notably, meta-analytic synthesis across animal studies suggests that glyphosate/GBHs often elicit sublethal physiological disruption in aquatic organisms even when dose–response patterns are not strictly monotonic, consistent with complex compensatory responses and stress thresholds [32]. In holothurians, rapid failure at the highest exposure may reflect the combination of (i) direct epithelial stress at key exchange surfaces, (ii) systemic redox overload, and (iii) destabilization of host–microbiota homeostasis, which together can precipitate rapid health decline [16,33,34,35].

### 4.3. Tissue Distribution Suggests Uptake via External Epithelia and Rapid Systemic Exposure

Glyphosate residues were detectable in multiple tissues, and the distribution pattern provided important clues regarding uptake and early internal transport in *A. japonicus*. Overall, the respiratory tree and coelomic fluid showed relatively higher glyphosate burdens than the body wall and intestine, suggesting rapid tissue-specific accumulation during the 24 h waterborne exposure period. This pattern is physiologically plausible because holothurians exchange solutes through the body surface and respiratory tree, whereas the coelomic fluid serves as an internal transport medium linking external exposure interfaces with systemic distribution.

At the medium exposure concentration, glyphosate levels in the respiratory tree were significantly higher than those in the other analyzed tissues, indicating that the respiratory tree may be an important uptake interface during early waterborne exposure. Under high-dose exposure, relatively elevated glyphosate levels were also detected in the respiratory tree and coelomic fluid, whereas the intestine did not consistently exhibit the highest residue burden. In the high-dose survivor group, the coelomic fluid showed the highest glyphosate level, while in the high-dose dead group, the respiratory tree and coelomic fluid remained among the main residue-bearing compartments. Together, these findings are more consistent with rapid uptake through external epithelial and respiratory surfaces, followed by systemic redistribution via the coelomic fluid, than with primary uptake through ingestion during this short exposure window [33,36,37].

Although the intestine is undoubtedly an important physiological target organ in deposit-feeding invertebrates such as *A. japonicus*, the present residue pattern suggests that, within the first 24 h of exposure, intestinal involvement may be secondary to uptake via external epithelial and respiratory interfaces. Overall, the tissue distribution pattern supports the interpretation that external epithelial and respiratory surfaces are likely major uptake routes of glyphosate during short-term waterborne exposure in *A. japonicus*. However, because the present study did not directly trace uptake pathways, this conclusion should be regarded as a physiologically plausible inference rather than definitive proof of route-specific uptake [4].

### 4.4. Oxidative Stress Is a Central Mechanism Linking Internal Dose to Functional Impairment

Oxidative responses were altered in a pathway-selective manner rather than as a uniform shift across all biomarkers. CAT activity decreased in the L, M, and HS groups; GSH-Px decreased in the HS group; and MDA increased in the L and M groups, whereas SOD, GR, and LZM did not differ significantly from the control. This pattern indicates that acute waterborne glyphosate exposure primarily affected peroxide-scavenging capacity and lipid peroxidation status, rather than causing a generalized disruption of the entire antioxidant and immune-related enzyme network. Given that oxidative stress is one of the most consistently reported mechanisms of glyphosate toxicity in aquatic organisms, the present results support redox imbalance as an important early event in the physiological response of *A. japonicus* to acute exposure [4,5].

The decrease in CAT across exposed groups, together with the reduction in GSH-Px in the HS group, is consistent with a compensation–depletion response under acute oxidative stress [37]. The elevated MDA levels in the L and M groups further indicate that lipid peroxidation had already occurred at sublethal exposure levels within the 24 h period. By contrast, MDA did not increase further in the highest-dose groups. This does not necessarily indicate the absence of oxidative stress; rather, under severe acute toxicity, rapid physiological impairment may limit the accumulation or detection of measurable lipid peroxidation products during a short exposure window. Mortality-associated collapse and increased between-individual variability in the high-dose groups may also contribute to this non-linear pattern.

Overall, the combined response of CAT, GSH-Px, and MDA is more consistent with a selective and non-linear oxidative stress phenotype than with a uniform dose-dependent increase across all biomarkers [4,5]. These findings suggest that oxidative stress is a central mechanism linking glyphosate exposure to subsequent functional impairment in *A. japonicus*, even though not all antioxidant or immune-related endpoints responded significantly within 24 h [32,36,37,38].

### 4.5. Digestive Enzyme Inhibition Implies Reduced Digestive Capacity and Energy Reallocation Under Stress

Among the assayed digestive enzymes, only trypsin activity decreased significantly across glyphosate-exposed groups, indicating a rapid perturbation of intestinal proteolytic capacity under acute waterborne exposure. In contrast, amylase (AMS) and ACP did not differ significantly from the control within the 24 h window, suggesting that carbohydrate-related digestion and ACP-linked processes were not measurably affected at this early time point. In sea cucumbers, reductions in digestive enzyme activities are commonly observed under chronic culture-related stressors (thermal, osmotic, nitrogenous wastes, and crowding) and are often coupled with oxidative stress and immune modulation, consistent with energy reallocation from growth/digestion toward maintenance and defense [34]. The present acute pattern therefore implies that waterborne glyphosate can compromise protein digestion rapidly, which may have disproportionate consequences for deposit feeders whose energy acquisition depends on efficient extracellular digestion and gut microbial support [36,39,40].

Mechanistically, the selective reduction in trypsin may arise from epithelial irritation, altered secretion dynamics, or oxidative damage to proteins and membranes in digestive tissues, even when other enzyme endpoints remain statistically unchanged. Rather than indicating a broad shutdown of intestinal function, these results support the view that proteolytic capacity may be an early-sensitive functional endpoint under acute chemical stress, potentially preceding more generalized digestive or immune impairment that may emerge under longer exposure duration or repeated stress [32,33,34].

### 4.6. Microbiome Dysbiosis Provides a Sensitive and Integrative Endpoint

The gut microbiome exhibited reduced alpha diversity (Shannon and Simpson) and clear compositional separation among groups (PCoA), indicating that glyphosate exposure rapidly restructured the intestinal community. Dysbiosis is increasingly considered a sensitive indicator in aquatic ecotoxicology because microbial communities respond quickly to chemical perturbation and can amplify host effects via barrier integrity, immune tone, and metabolic signaling [16,17,33,41].

LEfSe analysis identified discriminant taxa across treatments, with Vibrio enriched in M and HD groups and Psychrobacter enriched in HD. In aquaculture contexts, Vibrio expansion is frequently linked to opportunistic infection risk and impaired gut homeostasis in marine invertebrates, and microbiota-mediated protection against Vibrio-associated epithelial injury has been experimentally demonstrated in sea cucumber systems [34,36]. Meanwhile, shifts in community structure toward stress-associated Proteobacteria lineages (including Vibrionaceae and Moraxellaceae-related groups) have been reported under multiple aquaculture stressors in *A. japonicus*, supporting the view that these taxa can function as dysbiosis signatures rather than simple bystanders [34].

Notably, glyphosate’s antimicrobial mode of action (inhibition of the shikimate pathway in microbes and plants) provides a plausible mechanistic basis for microbiome restructuring, with downstream consequences for host nutrition and immunity; this concept is consistent with the microbiota–host axis literature and with aquatic fish studies showing that glyphosate-driven dysbiosis can disrupt enterohepatic signaling and induce gut inflammation and barrier damage [33,41]. Therefore, the observed PCoA separation and taxonomic biomarkers likely reflect an integrated host–microbe response that connects chemical exposure to functional outcomes (digestive enzyme inhibition and immune weakening) [33,34,35].

### 4.7. Implications for Aquatic Ecotoxicology and Sea Cucumber Aquaculture

Together, the residue distribution, oxidative stress phenotype, digestive suppression, and microbiome dysbiosis support a coherent “stress signature” of waterborne glyphosate exposure in *A. japonicus*. From an ecotoxicology perspective, these endpoints complement traditional survival-based metrics by providing mechanistic markers that may respond at lower concentrations and earlier time points, improving hazard identification and comparative sensitivity analysis across taxa [33]. From an aquaculture standpoint, the results highlight that short-term water quality deterioration associated with herbicide inputs could elevate disease susceptibility (via oxidative/immune disruption) while simultaneously reducing digestive capacity and altering microbiota stability, a combination that may reduce resilience even if mortality is not immediately observed [34,39,40].

Given that coastal aquaculture often co-occurs with agricultural watersheds, integrating chemical monitoring (measured exposure confirmation), oxidative biomarkers (CAT, GSH-Px, MDA; with SOD/GR as supportive indices), digestive enzymes (trypsin; with amylase/ACP as supportive indices), and microbiome indices (alpha diversity, PCoA, key opportunists such as Vibrio) could provide a practical framework for early-warning surveillance in sea cucumber farming areas [34,36].

### 4.8. Limitations and Future Directions

This study used acute exposure (24 h), and longer-term work is needed to determine whether the observed microbiome changes persist, whether digestive capacity recovers, and how these endpoints relate to growth, immune competence, and disease outcomes under realistic multi-stressor culture conditions (temperature and salinity fluctuations, hypoxia, nitrogenous wastes) [34,39]. Future experiments should also quantify AMPA alongside glyphosate in tissues and explore whether microbiome modulation is driven primarily by direct antimicrobial selection, indirect host physiology shifts (e.g., oxidative and immune status), or both [33,41]. Finally, differentiating pure glyphosate from commercial formulations is important for risk interpretation, as formulation additives can alter toxicity profiles in aquatic organisms [32,35].

## 5. Conclusions

This study characterized acute physiological stress responses in the sea cucumber *Apostichopus japonicus* under waterborne glyphosate exposure. Short-term exposure resulted in mortality at the highest dose and was associated with rapid internal distribution of glyphosate, selective oxidative imbalance, reduced intestinal trypsin activity, and gut microbiota restructuring. These results indicate that waterborne glyphosate can rapidly impair physiological homeostasis in this mariculture-relevant species and highlight the value of integrating biochemical biomarkers and microbiome responses in aquatic ecotoxicology.

## Data Availability

The original contributions presented in this study are included in the article. Further inquiries can be directed to the corresponding authors.

## References

[1] Duke S.O., Powles S.B. (2008). Glyphosate: A once-in-a-century herbicide. Pest Manag. Sci..

[2] Klátyik S., Simon G., Oláh M., Takács E. (2024). Aquatic ecotoxicity of glyphosate, its formulations, and co-formulants: Evidence from 2010 to 2023. Environ. Sci. Eur..

[3] Duke S.O. (2018). The history and current status of glyphosate. Pest Manag. Sci..

[4] Rodríguez-Gil J.L., Prosser R.S., Duke S.O., Solomon K.R. (2021). Ecotoxicology of Glyphosate, Its Formulants, and Environmental Degradation Products. Rev. Environ. Contam. Toxicol..

[5] Carretta L., Masin R., Zanin G. (2022). Review of studies analysing glyphosate and aminomethylphosphonic acid (AMPA) occurrence in groundwater. Environ. Rev..

[6] Álvarez Bayona M.A., Maturana Córdoba A., Gallardo Amaya R.J., Muñoz Acevedo A. (2022). Occurrence of glyphosate in surface and drinking water sources in Cúcuta, Norte de Santander, and its removal using membrane technology. Front. Environ. Sci..

[7] Tresnakova N., Stara A., Velisek J. (2021). Effects of Glyphosate and Its Metabolite AMPA on Aquatic Organisms. Appl. Sci..

[8] European Food Safety Authority (EFSA) (2023). Peer review of the pesticide risk assessment of the active substance glyphosate. EFSA J..

[9] United States Environmental Protection Agency (US EPA) (2022). EPA Withdraws Glyphosate Interim Decision. https://www.epa.gov/pesticides/epa-withdraws-glyphosate-interim-decision.

[10] International Agency for Research on Cancer (IARC) (2017). Some Organophosphate Insecticides and Herbicides. IARC Monographs on the Evaluation of Carcinogenic Risks to Humans.

[11] Rendón-von Osten J., Borges-Ramírez M.M., Ruiz-Velazco N.G., Helguera E., Arellano-Aguilar O., Peregrina-Lucano A.A., Lozano-Kasten F. (2025). Glyphosate and AMPA in Groundwater, Surface Water, and Soils Related To Different Types of Crops in Mexico. Bull. Environ. Contam. Toxicol..

[12] Li Z.-M., Kannan K. (2025). Occurrence and Distribution of Glyphosate and Aminomethylphosphonic Acid in Surface Water, Drinking Water, Wastewater and Other Water Types from New York State, USA. Water Res..

[13] Wang S., Liu B., Yuan D., Ma J. (2016). A simple method for the determination of glyphosate and aminomethylphosphonic acid in seawater matrix with high performance liquid chromatography and fluorescence detection. Talanta.

[14] Nedelkoska T.V., Low G.K.-C. (2004). High-performance liquid chromatographic determination of glyphosate in water and plant material after pre-column derivatisation with 9-fluorenylmethyl chloroformate. Anal. Chim. Acta.

[15] Alonso B., Griffero L., Bentos Pereira H., Pareja L., Pérez Parada A. (2022). Determination of glyphosate and AMPA in freshwater and soil from agroecosystems by 9-fluorenylmethoxycarbonyl chloride derivatization and liquid chromatography-fluorescence detection and tandem mass spectrometry. MethodsX.

[16] Huang Y., Huang Q., Zhou K., Luo X., Long W., Yin Z., Huang Z., Hong Y. (2024). Effects of glyphosate on neurotoxicity, oxidative stress and immune suppression in red swamp crayfish, Procambarus clarkii. Aquat. Toxicol..

[17] Ribeiro Y.M., Moreira D.P., Weber A.A., Sales C.F., Melo R.M.C., Bazzoli N., Rizzo E., Paschoalini A.L. (2022). Adverse effects of herbicides in freshwater Neotropical fish: A review. Aquat. Toxicol..

[18] Mazuryk J., Klepacka K., Kutner W., Sharma P.S. (2024). Glyphosate: Impact on the microbiota-gut-brain axis and the immune-nervous system, and clinical cases of multiorgan toxicity. Ecotoxicol. Environ. Saf..

[19] Gallardo-Gómez Ú., Juárez-Jiménez B., Correa-Galeote D., Zafra-Gómez A. (2025). Sea cucumbers as bioindicators of pollution and sea cucumber microbiomes as markers of environmental stress: A review. J. Environ. Chem. Eng..

[20] Yu J., Sawabe T., Yamano R., Koike S., Sakai Y., Mino S. (2023). Inferring potential causative microbial factors of intestinal atrophic disease in the sea cucumber *Apostichopus japonicus*. Front. Mar. Sci..

[21] Zamora L.N., Yuan X., Carton A.G., Slater M.J. (2018). Role of deposit-feeding sea cucumbers in integrated multitrophic aquaculture: Progress, problems, potential and future challenges. Rev. Aquac..

[22] Mohsen M., Ismail S., Yuan X., Yu Z., Lin C., Yang H. (2024). Sea cucumber physiological response to abiotic stress: Emergent con-taminants and climate change. Sci. Total Environ..

[23] Cui L., Xie Y., Luo K., Wang M., Liu L., Li C., Tian X. (2024). Physiological and intestinal microbiota responses of sea cucumber *Apostichopus japonicus* to various stress and signatures of intestinal microbiota dysbiosis. Front. Microbiol..

[24] Huo D., Su F., Zhang L., Yang H., Sun L. (2022). Temperature and dissolved oxygen influence the immunity, digestion, and antioxidant level in sea cucumber *Apostichopus japonicus*. Front. Mar. Sci..

[25] European Food Safety Authority (EFSA) (2015). Conclusion on the peer review of the pesticide risk assessment of the active substance glyphosate. EFSA J..

[26] U.S. Environmental Protection Agency (EPA) (2020). Glyphosate: Interim Registration Review Decision Case Number 0178. https://www.epa.gov/ingredients-used-pesticide-products/glyphosate.

[27] Zhao J., Chen J., Tian X., Jiang L., Cui Q., Sun Y., Wu N., Liu G., Ding Y., Wang J. (2023). Amantadine Toxicity in *Apostichopus japonicus* Revealed by Proteomics. Toxics.

[28] Tian X., Li H., Zhang X., Xu Y., Zhang H., Han D., Liu H., Wang B., Cui Y., Liu H. (2021). Effects of Acute and Chronic Exposure to Semicarbazide on the Sea Cucumber *Apostichopus japonicus*. Front. Environ. Sci..

[29] Wang L., Pei H., Xing T., Chen D., Chen Y., Hao Z., Tian Y., Ding J. (2025). Gut bacteria and host metabolism: The keys to sea cucumber (*Apostichopus japonicus*) quality traits. Food Chem..

[30] Chen D., Pei H., Chen Y., Liu A., Xing T., Zhang H., Wang L. (2025). Dietary Glycine and Methyl Donors Remodel Gut Microbiota to Enhance Collagen Synthesis in Sea Cucumber (*Apostichopus japonicus*). Biology.

[31] (2007). Determination of Glyphosate Residues in Food for Import and Export—HPLC-MS/MS Method.

[32] Mohsen M., Chenggang L., Sui Y., Yang H. (2023). Fate of Microplastic Fibers in the Coelomic Fluid of the Sea Cucumber *Apostichopus japonicus*. Environ. Toxicol. Chem..

[33] Yan B., Sun Y., Fu K., Zhang Y., Lei L., Men J., Guo Y., Wu S., Han J., Zhou B. (2023). Effects of glyphosate exposure on gut-liver axis: Metabolomic and mechanistic analysis in grass carp (*Ctenopharyngodon idellus*). Sci. Total Environ..

[34] Zhao Y., Jia C., Gao F., Zhang H. (2024). Gut microbiota of sea cucumbers, with a focus on *Apostichopus japonicus*. The World of Sea Cucumbers.

[35] Moraes J.S., Ballesteros M.L., Hued A.C., Bonifacio A.F., Azambuja T.G., Vaz B.D.S., Martins C.M.G. (2024). Glyphosate and its formulated product Roundup Transorb R® affect locomotor activity and reproductive and developmental parameters in Jenynsia lineata fish: An intergenerational study. Chemosphere.

[36] Iwalaye O.A., Moodley G.K., Robertson-Andersson D.V. (2020). The possible routes of microplastics uptake in sea cucumber Holothuria cinerascens (Brandt, 1835). Environ. Pollut..

[37] Zhu M., Wang Z., Chen J., Xie H., Zhao H., Yuan X. (2020). Bioaccumulation, Biotransformation, and Multicompartmental Toxicokinetic Model of Antibiotics in Sea Cucumber (*Apostichopus japonicus*). Environ. Sci. Technol..

[38] Drewek A., Lubawy J., Domek P., Polak J., Słocińska M., Dzięgelewska A., Klimaszyk P. (2024). Behavioral and Biochemical Effects of Glyphosate-Based Herbicide Roundup on Unionid Mussels: Are Mussels Good Indicators of Water Pollution with Glyphosate-Based Pesticides?. Water.

[39] Sun Y., Wang S., Wang C., Wang M., Kang W., Qu L., Song J., Zhao C., Wang Q. (2024). Impact of Starfish Predatory Pressure on the Immune and Antioxidant Functions of Sea Cucumber *Apostichopus japonicus*. Fishes.

[40] Song M., Zhang S., Xiao K., Zhang X., Li C. (2024). Intestinal microbiota protects Vibrio splendidus-induced intestinal epithelium damage by inhibiting apoptosis and promoting proliferation in *Apostichopus japonicus*. Aquaculture.

[41] Matsuzaki R., Gunnigle E., Geissen V., Clarke G., Nagpal J., Cryan J.F. (2023). Pesticide exposure and the microbiota-gut-brain axis. ISME J..

